# Hypertensive Disorders of Pregnancy and Breastfeeding Among US Women

**DOI:** 10.1001/jamanetworkopen.2025.21902

**Published:** 2025-07-18

**Authors:** Deanna Nardella, Maureen E. Canavan, Sarah N. Taylor, Mona Sharifi

**Affiliations:** 1 Department of Pediatrics, Yale University School of Medicine, New Haven, Connecticut; 2 Yale Cancer Outcomes, Public Policy and Effectiveness Research Center, Yale School of Medicine, New Haven, Connecticut

## Abstract

**Importance:**

Hypertension contributes to US maternal-infant morbidity and mortality, with potential attenuation from breastfeeding. Little is known regarding breastfeeding outcomes among mother-infant dyads exposed to hypertensive disorders of pregnancy (HDP).

**Objective:**

To quantify the extent to which HDP is associated with never breastfeeding and the time to breastfeeding cessation among postpartum women in the US.

**Design, Setting, and Participants:**

This cross-sectional study used nationally representative data from the Centers for Disease Control and Prevention Pregnancy Risk Assessment Monitoring System (PRAMS). Participants included women from 43 US states, Washington, DC, and Puerto Rico who had a live birth between January 2016 and November 2022. Complete data for HDP exposure, outcomes of breastfeeding noninitiation or cessation, and all covariates were included in the analysis, performed from October to December 2024.

**Exposure:**

Self-reported high blood pressure or hypertension, preeclampsia, or eclampsia before or during pregnancy.

**Main Outcomes and Measures:**

Odds of never breastfeeding, hazard of breastfeeding cessation, and median time to breastfeeding cessation, adjusted for sociodemographic and maternal-infant health covariates.

**Results:**

Of 205 247 women (weighted number, 10 915 302), mean (SD) age was 30.0 (5.8) years; 99.0% were first-time mothers; 54.0% had private and 40.0% had Medicaid insurance; and 91.0% delivered a term infant. HDP and breastfeeding initiation were reported by approximately 17.0% and 88.0% of women, respectively. Adjusting for covariates, HDP was associated with higher odds of never breastfeeding (adjusted odds ratio, 1.11; 95% CI, 1.05-1.18), and among those who breastfed, a higher adjusted hazard of breastfeeding cessation (adjusted hazard ratio, 1.17; 95% CI, 1.14-1.21). The median time to breastfeeding cessation among those with HDP was 17 weeks shorter than among those without HDP (unadjusted median duration, 17 [IQR, 5.0 to >46.7] vs 34 [IQR, 9.0 to >46.7] weeks).

**Conclusions and Relevance:**

In this cross-sectional study representing more than 10 million US postpartum women, HDP was associated with higher odds of never breastfeeding and a higher hazard of breastfeeding cessation. Studies to understand the potential mechanisms of this association are required to develop targeted breastfeeding support strategies for individuals with HDP.

## Key Points

**Question:**

Are hypertensive disorders of pregnancy (HDP) associated with breastfeeding initiation and duration?

**Findings:**

In this cross-sectional study representing over 10 million US women, HDP was associated with higher odds of never breastfeeding. Among those who initiated breastfeeding, HDP was associated with a higher hazard of breastfeeding cessation and fewer weeks of breastfeeding.

**Meaning:**

These findings suggest that postpartum women with HDP may benefit from tailored interventions to promote the cardioprotective benefits of breastfeeding.

## Teaser

This cross-sectional study examines the extent to which exposure to hypertensive disorders of pregnancy is associated with never breastfeeding and, for those who initiate breastfeeding, the time to breastfeeding cessation among a national sample of postpartum US women.

## Introduction

Mortality rates for women and infants in the US are rising,^1,2,3^ are among the highest in high-income nations,^1,2,3,4^ and disproportionately impact communities that are socially and/or economically marginalized.^5,6,7,8,9^ Hypertensive disorders of pregnancy (HDP), including chronic or gestational hypertension, preeclampsia, and eclampsia, are top drivers of US maternal and infant morbidity and mortality.^1,7,9^ The incidence of HDP more than doubled between 1993 and 2017.^10^ From 2017 to 2019, 16% of US births were complicated by HDP, and 32% of maternal deaths during delivery hospitalizations included an HDP diagnosis.^1,7^ HDP exposure increases women’s long-term risk for heart disease (eg, myocardial infarctions, ischemic heart disease, venous thromboembolism), end-stage kidney disease, and stroke.^11,12,13,14,15^ Additionally, the all-cause mortality rate for US infants increased in 2022 for the first time in 2 decades, with a 9% increase in infant death secondary to maternal complications.^9^ Beyond peripartum mortality risk, HDP exposure is associated with adverse long-term cardiometabolic outcomes among women and their offspring.^16,17,18,19^

Breastfeeding may mitigate the risks associated with HDP for mother-infant dyads who are exposed.^20^ Prior studies have observed an association between breastfeeding and lower long-term risk of maternal cardiometabolic disease,^21,22,23,24,25,26,27,28,29^ preeclampsia,^30^ and both maternal and infant all-cause mortality.^22,31,32^ A dose-dependent association has been observed, with longer breastfeeding durations associated with lower cardiometabolic risk for both infants^33,34,35,36^ and women,^22,24,29,37^ with potential for augmented cardiovascular benefit among those with HDP compared with those without.^29^

Despite 83% of US women initiating breastfeeding at delivery, only 25% exclusively breastfeed through 6 months^38^ as recommended by leading health organizations,^39,40,41,42,43,44^ highlighting the presence of multifactorial barriers to sustaining exclusive breastfeeding faced by families. Though other maternal comorbidities, including gestational diabetes,^45,46,47,48,49,50,51^ have been seen to negatively influence breastfeeding outcomes, little is known about breastfeeding outcomes for the 16% of US pregnant women with HDP compared with women without HDP.^1,6,38,52,53^ A few observational studies have found worse breastfeeding outcomes for women with HDP,^29,54,55,56,57,58^ with most of these studies conducted outside the US,^29,54,55^ apart from 2 studies that examined women from single US states with data collected nearly 1 decade prior (2004-2015).^56,57^ Other studies considered only preterm deliveries^57^ and presented breastfeeding duration as any breastfeeding longer than 8 weeks post partum.^56,57^

Understanding the association between HDP exposure and breastfeeding outcomes could inform efforts to promote more equitable access to the maternal-infant cardioprotective benefits of breastfeeding and more equitable health outcomes amid rising rates of maternal and infant cardiometabolic disease and mortality.^1,2,6,9,21,22,23,24,26,31,33^ We aimed to quantify the extent to which HDP exposure is associated with never breastfeeding and, for those who initiate breastfeeding, the hazard of and median time to breastfeeding cessation among a national sample of US women who were surveyed by the Centers for Disease Control and Prevention (CDC) between January 2016 and November 2022. We hypothesized that women with vs without HDP would have a higher probability of both never breastfeeding and earlier breastfeeding cessation.

## Methods

We conducted a serial cross-sectional analysis with stratified, weighted data from the Centers for Disease Control and Prevention Pregnancy Risk Assessment Monitoring System (PRAMS). In brief, PRAMS contacts US women at a single time point between 2 and 6 months post partum (mean, 4 months), surveying them on their attitudes, beliefs, and experiences over the prepregnancy, prenatal, and postpartum periods.^59,60^ Each US jurisdiction samples 1000 to 3000 women annually, oversampling from smaller sociodemographic populations and assigning sample weights to each response to best reflect the demographic characteristics of that jurisdiction.^59^ We use the term *women* to describe our study sample to align with terminology used by PRAMS.

The Yale University Institutional Review Board deemed this study exempt from human participant research review and informed consent. We report our findings per Strengthening the Reporting of Observational Studies in Epidemiology (STROBE) guidelines.

### Study Population, Data Collection, and Measures

Our analytic sample included women who had a live infant at survey completion and complete data for HDP, with noninitiation or cessation of breastfeeding, and with maternal-infant sociodemographic and health covariates based on prior studies examining breastfeeding outcomes with PRAMS data.^61,62^ All participants delivered their infant(s) between 2016 and 2021 and completed the PRAMS survey between January 2016 and November 2022. The primary dependent variables were never breastfeeding and time to breastfeeding cessation (in weeks). Women were determined to have never breastfed if they reported “no” to ever breastfeeding. Among those who reported breastfeeding, we defined time to breastfeeding cessation as 0.5 weeks if they reported breastfeeding less than 1 week (4.3%), their exact breastfeeding duration (in weeks) if provided by women (35.3%), or infant age at the time of survey completion (in weeks) if women responded, “still breastfeeding” (60.4%).

HDP, our primary independent variable, was defined as self-reported high blood pressure or hypertension, preeclampsia, or eclampsia before or during pregnancy (yes or no). Several maternal-infant sociodemographic and health covariates were included based on available variables, prior associations observed with breastfeeding in the literature, or clinical relevance.^61,62^

Sociodemographic covariates included maternal age (<18, 18-24, 25-29, 30-34, or >35 years), maternal insurance at the time of delivery (Medicaid, private, self-pay, or other), household income (<$20 000, $20 001-$40 000, $40 001-$60 000, $60 001-$85 000,≥$85 000, or not reported), race and ethnicity (detailed below), enrollment in the Women, Infants and Children (WIC) supplemental assistance program during pregnancy (enrolled or not enrolled), marital status (married or not married), and educational level (≤12 or >12 years).^61,62^ PRAMS reports race and ethnicity based on birth certificate files used to draw the stratified sample. We defined a combined race and ethnicity variable to reflect well-described differences in US breastfeeding initiation and duration rates and align with 2024 federal guidelines for standard reporting of race and ethnicity.^63,64,65,66^ The categories are as follows: American Indian or Alaska Native, Asian or Pacific Islander (including Chinese, Filipino, Hawaiian, Japanese, or Other Asian), non-Hispanic Black, non-Hispanic White, non-Hispanic multiracial, or other. Hispanic ethnicity was assigned to participants if reported; otherwise, individuals’ races were assigned. The category other reflects those individuals reported as “Other, non-White” on the infant’s birth certificate and did not report Hispanic ethnicity. Additionally, a single categorical level was made for Asian or Pacific Islander given small sample sizes for Chinese (1.3%), Filipino (0.9%), Hawaiian (0.1%), Japanese (0.3%), and Other Asian (5.0%) racial and ethnic groups.

Maternal health covariates included bivariate variables for first child, singleton pregnancy, premature delivery (gestational age, <37 weeks), body mass index (calculated as the weight in kilograms divided by the height in meters squared) of 30 or greater, any smoking reported in pregnancy, cesarean delivery, and a pregestational or gestational diagnosis of diabetes and/or depression. Infant health covariates included sex (female or male), birth weight (very low, <1500 g; low, 1500 to <2500 g; normal, 2500 to <4000 g; or large, ≥4000 g), and length of hospital stay (<3, 3-5, or ≥6 days).

### Statistical Analysis

Data were analyzed from October to December 2024. We summarized the characteristics of the study sample and the prevalence of HDP by sociodemographic and maternal-infant health covariates and reported statistically significant differences using χ^2^ tests. We performed multivariable logistic regression, including all sociodemographic and maternal-infant health covariates selected a priori in our model, reporting odds ratios (ORs) of never breastfeeding by HDP exposure.^67^ Reference groups reflect those used in prior literature and reflect subgroups previously associated with higher rates of breastfeeding duration.^61,62^ A 2-sided *P* < .05 was used for a threshold of statistical significance.

We performed a multivariable Cox proportional hazards regression, controlling for all covariates, and calculated unadjusted Kaplan-Meier estimates, reporting hazard ratios (HRs) for breastfeeding cessation by HDP exposure and the median time to breastfeeding cessation (weeks post partum). Reports of still breastfeeding at the time of survey completion were considered censored events in our Cox proportional hazards model (ie, participants did not self-report the event of breastfeeding cessation). Women who were still breastfeeding were assigned their infant’s age at the time of survey completion as their known breastfeeding duration. The proportional hazards assumption was violated for race and ethnicity. Thus, our results for this covariate reflect a mean rather than an instantaneous hazard of breastfeeding cessation at any specific time point within our analytical timeframe.

Given well-described inequities in HDP and breastfeeding rates between US racial and ethnic groups, we tested for potential effect modification by race and ethnicity on the association between HDP and both breastfeeding initiation and cessation. Stratified analyses were completed for those models found to have significant interaction terms, adjusting for all other covariates included within our larger models. We used Stata, version 17.0 (StataCorp LLC), to account for complex survey design and weighting.

## Results

Our analytic sample used to complete our multivariable logistic regression included 205 247 women (weighted number, 10 915 302) from 43 US states, Washington, DC, and Puerto Rico with a mean (SD) age of 30.0 (5.8) years. In terms of race and ethnicity, 0.8% were American Indian or Alaska Native; 5.3%, Asian or Pacific Islander; 15.0%, Black; 18.0%, Hispanic; 50.8%, White; 2.3% multiracial; and 0.7%, other. Most of the sample were first time mothers (99.0%), with private (54.0%) or Medicaid (40.0%) insurance, and delivered at term (91.0%). All participants gave birth between 2016 and 2021. The mean (SD) infant age at survey completion was 18.2 (5.3) weeks (range 9.0-46.7 weeks). Table 1 displays the characteristics of the weighted sample overall and the prevalence of HDP by several maternal-infant covariates. For our multivariable Cox proportional hazards regression analysis, we included the 88.0% of women in the analytic sample who reported ever breastfeeding. Mean (SD) infant age at survey completion was 18.1 (5.2) weeks. Of respondents, 0.2% completed the survey by 10 weeks post partum, 68.3% by 20 weeks, 96.9% by 30 weeks, and 99.9% by 40 weeks. The latest survey was completed at 46.7 weeks post partum (Table 2).

**Table 1.** Characteristics of Study Sample and Prevalence of HDP by Maternal-Infant Covariates^a^

Characteristic	Total sample, No. (column %)^b^^,^^c^	With HDP diagnosis, No. (row %)^b^^,^^d^	*P* value^e^
Maternal age, y			
<18	2441 (1.1)	452 (18.0)	<.001
18-24	43 936 (21.0)	8143 (16.0)	
25-29	58 847 (29.0)	10 809 (16.0)	
30-34	61 332 (30.0)	11 576 (16.0)	
≥35	38 691 (19.0)	8821 (19.0)	
Insurance at delivery			
Medicaid	88 114 (40.0)	17 860 (18.0)	<.001
Private	104 343 (54.0)	19 707 (16.0)	
Self-pay	4808 (2.4)	697 (11.0)	
Other	7982 (3.4)	1537 (15.0)	
Income, US $			
<20 000	52 571 (23.0)	10 892 (18.0)	<.001
20 001-40 000	39 999 (19.0)	8112 (18.0)	
40 001-60 000	24 242 (12.0)	4880 (17.0)	
60 001-85 000	20 756 (10.0)	4040 (17.0)	
>85 000	8897 (28.0)	8897 (15.0)	
Not reported	2980 (8.2)	2980 (15.0)	
Race and ethnicity^f^			
American Indian or Alaska Native	8144 (0.8)	1678 (21.0)	<.001
Asian or Pacific Islander	14 232 (5.3)	1685 (10.0)	
Black	36 143 (15.0)	9111 (23.0)	
Hispanic	40 326 (18.0)	6311 (13.0)	
White	95 328 (58.0)	18 861 (17.0)	
Multiracial	9904 (2.3)	1999 (18.0)	
Other	1170 (0.7)	156 (10.0)	
WIC enrollment			
Enrolled	129 162 (66.0)	24 374 (16.0)	<.001
Not enrolled	76 085 (34.0)	15 427 (18.0)	
Marital status			
Married	122 869 (62.0)	22 101 (15.0)	<.001
Not married	82 378 (38.0)	17 700 (19.0)	
Educational level, y			
≤12	72 758 (35.0)	14 451 (17.0)	<.001
>12	132 489 (65.0)	25 350 (16.0)	
First child			
Yes	201 729 (99.0)	38 681 (17.0)	<.001
No	3518 (0.9)	1120 (30.0)	
Singleton pregnancy			
Yes	198 374 (98.0)	37 643 (16.0)	<.001
No	6873 (1.7)	2158 (30.0)	
Infant birth weight, g			
<1500 (very low)	5484 (1.0)	2247 (42.0)	<.001
<2500 (low)	35 156 (6.0)	10 434 (29.0)	
2500-4000 (normal)	150 560 (85.0)	19127 (12.0)	
>4000 (large)	13 935 (8.0)	1410 (9.7)	
Infant sex			
Female	103 036 (51.0)	16 503 (14.0)	.72
Male	102 099 (49.0)	16 715 (14.0)	
Premature delivery (gestational age)			
Yes (<37 wk)	34 683 (8.6)	11 586 (31.0)	<.001
No (≥37 wk)	170 452 (91.0)	21 632 (12.0)	
Infant hospital length of stay, d			
<3	112 205 (61.0)	11 842 (10.0)	<.001
3-5	63 878 (32.0)	11 556 (16.0)	
>5	29 052 (7.6)	9820 (31.0)	
Maternal BMI			
<30	144 185 (71.0)	17 937 (10.0)	<.001
≥30	60 950 (29.0)	15 281 (22.0)	
Smoking status			
Smoker	15 282 (6.5)	2804 (16.0)	<.001
Nonsmoker	189 853 (94.0)	30 414 (13.0)	
Diabetes before or during pregnancy			
Yes	26 968 (12.0)	7218 (24.0)	<.001
No	178 167 (88.0)	26 000 (12.0)	
Depression before or during pregnancy			
Yes	20 847 (9.3)	4891 (21.0)	<.001
No	184 288 (91.0)	28 327 (13.0)	
Cesarean section delivery			
Yes	68 495 (31.0)	15 599 (18.0)	<.001
No	136 640 (69.0)	17 619 (11.0)	

Abbreviations: BMI, body mass index (calculated as the weight in kilograms divided by the height in meters squared); HDP, hypertensive disorders of pregnancy; WIC, Women, Infants and Children supplemental assistance program.

^a^ Analysis include Pregnancy Risk Assessment Monitoring System complex survey design and weighting. US states and territories included in this analysis are Alabama, Alaska, Arkansas, Arizona, Colorado, Connecticut, Delaware, Florida, Georgia, Hawaii, Illinois, Indiana, Iowa, Kansas, Kentucky, Louisiana, Maine, Massachusetts, Maryland, Michigan, Minnesota, Mississippi, Missouri, Montana, Nebraska, New Hampshire, New Jersey, New Mexico, New York, New York City, North Carolina, North Dakota, Oklahoma, Pennsylvania, Puerto Rico, Rhode Island, South Dakota, Tennessee, Texas, Utah, Virginia, West Virgina, Washington, DC, Wisconsin, West Virgina, and Wyoming.

^b^ Raw totals and weighted percentages are presented. Columns may not add to 100 due to rounding.

^c^ Includes 205 247 women (weighted number, 10 915 302).

^d^ Includes 39 801 women (19.4%; weighted number, 1 815 717 [16.6%]).

^e^ Calculated using χ^2^ tests comparing each maternal-infant covariate level by HDP.

^f^ All race and ethnicity information are from birth certificate data. Other includes individuals reported as Other, non-White, and who did not report Hispanic ethnicity.

**Table 2.** Odds of Never Breastfeeding and Hazard of Breastfeeding Cessation Among US Women With vs Without HDP, 2016-2022^a^

Diagnosis of HDP	Odds of never breastfeeding^b^^,^^c^		Hazard of breastfeeding cessation^b^^,^^d^^,^^e^	
	AOR (95% CI)	*P* value	AHR (95% CI)	*P* value
Yes	1.11 (1.05-1.18)	<.001	1.17 (1.14-1.21)	<.001
No	1 [Reference]	NA	1 [Reference]	NA

Abbreviations: AHR, adjusted hazard ratio; AOR, adjusted odds ratio; HDP, hypertensive disorders of pregnancy; NA, not applicable.

^a^ Analysis include Pregnancy Risk Assessment Monitoring System complex survey design and weighting. US states and territories included in this analysis are Alabama, Alaska, Arkansas, Arizona, Colorado, Connecticut, Delaware, Florida, Georgia, Hawaii, Illinois, Indiana, Iowa, Kansas, Kentucky, Louisiana, Maine, Massachusetts, Maryland, Michigan, Minnesota, Mississippi, Missouri, Montana, Nebraska, New Hampshire, New Jersey, New Mexico, New York, New York City, North Carolina, North Dakota, Oklahoma, Pennsylvania, Puerto Rico, Rhode Island, South Dakota, Tennessee, Texas, Utah, Virginia, West Virgina, Washington, DC, Wisconsin, West Virgina, and Wyoming.

^b^ All models are adjusted for maternal age; insurance at delivery; income; race and ethnicity; marital status; educational level; Women, Infants and Children enrollment; first child; single vs multiple gestation; cesarean delivery; maternal BMI, depression, diabetes, and smoking; and infant sex, prematurity, length of hospital stay, and birth weight.

^c^ Adjusted logistic model; includes 205 247 women (weighted number, 10 915 302).

^d^ Percentage of total analytical sample who had completed the survey by time point post partum: 0.2% by 10 weeks, 68.3% by 20 weeks, 96.9% by 30 weeks, and 99.9% by 40 weeks. The latest time of survey completion was 46.7 weeks post partum.

^e^ Adjusted Cox proportional hazards model; includes 180 660 women (weighted number, 9 570 975).

Seventeen percent of women reported an HDP diagnosis. HDPs were most common among women younger than 18 years or 35 years or older, of American Indian or Alaska Native and non-Hispanic Black race and ethnicity, not married, insured by Medicaid, and with household income of $40 000 or less. Women with HDPs were more likely to have a body mass index of 30 or greater, to have a pregestational or gestational diagnosis of diabetes or depression, to smoke during pregnancy, and to have a multiple gestation or cesarean delivery. Infants of women with HDPs were more likely to be premature, have a low or very low birth weight, and be admitted to the hospital for more than 5 days post delivery (Table 1).

We present a complete case analysis and assume data to be missing at random. All covariates included in our adjusted models had less than 5% missing values, outside of income (8.2% missing), for which unreported was included. Overall, 10% of observations were removed for missing data. To assess for response bias, the eTable in Supplement 1 compares nonparticipants with participants by all covariates included in our model. Raw frequency values between comparable covariate levels differed by 5% or more for private insurance (5.2%), self-pay insurance (6.2%), and non-Hispanic White race or ethnicity (5.1%).

### HDP and Odds of Never Breastfeeding

The prevalence of breastfeeding initiation was 85% among women with HDP and 88% among those without. Adjusting for all sociodemographic and maternal-infant characteristics above, women with an HDP had higher odds of never breastfeeding (adjusted OR, 1.11; 95% CI, 1.05-1.18; *P* < .001) (Table 2). There were no significant interactions found between race and ethnicity and the association between HDP and breastfeeding initiation.

### HDP and Postpartum Breastfeeding Cessation

**Figure 1.**
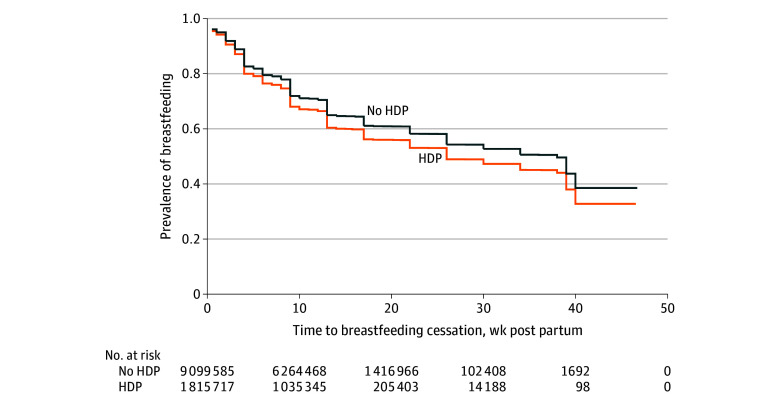
Time to Breastfeeding Cessation Among Women With vs Without Hypertensive Disorders of Pregnancy (HDP) The graphs demonstrate the time to breastfeeding cessation (0-40 weeks) for individuals with and without HDP among a sample of women who delivered a liveborn infant in the US between 2016 and 2021.

**Figure 2.**
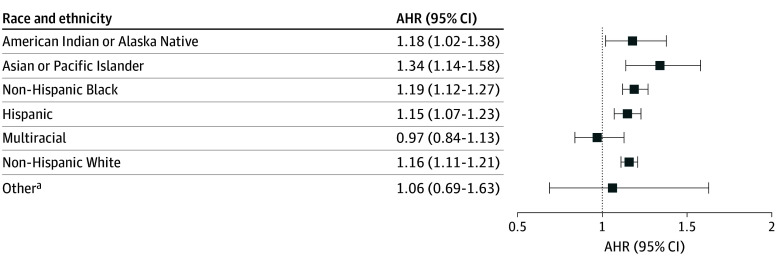
Hazard of Breastfeeding Cessation by Race and Ethnicity Data are stratified by exposure to hypertensive disorders of pregnancy (HDP) and race and ethnicity among women who delivered a liveborn infant between 2016 and 2021 in the US. AHR indicates adjusted hazard ratio. ^a^Other reflects those individuals reported as “Other, non-White” on the infant’s birth certificate and who did not report Hispanic ethnicity.

Figure 1 displays adjusted Cox survival curves demonstrating the time to breastfeeding cessation for individuals with and without HDP. Among participants who had initiated breastfeeding, HDP was associated with a higher hazard of breastfeeding cessation in our adjusted model (adjusted HR, 1.17; 95% CI, 1.14-1.21). Women with HDP had a median time to breastfeeding cessation of 17 (IQR, 5.0 to >46.7) weeks compared with 34 (IQR, 9.0 to >46.7) weeks for women without HDP. Note that an upper quartile range was not obtained, as more than 25% of women in our sample were still breastfeeding at the time of last observation (46.7 weeks). We observed statistically significant interactions between race and ethnicity and the association between HDP and breastfeeding cessation (non-Hispanic Black participants, *P* = .049; Hispanic participants, *P* = .02; multiracial participants, *P* = .01). In our fully adjusted model stratified by race and ethnicity, we found the highest hazard of breastfeeding cessation among Asian or Pacific Islander (adjusted HR, 1.34; 95% CI, 1.14-1.58), non-Hispanic Black (adjusted HR, 1.19; 95% CI, 1.12-1.27), and American Indian or Alaska Native (adjusted HR, 1.18; 95% CI, 1.02-1.38) women with HDP (Figure 2).

## Discussion

In this cross-sectional analysis of nationally representative data from US women, exposure to HDP was associated with 11% higher odds of never breastfeeding and, among those who do initiate breastfeeding, a 17% higher hazard of breastfeeding cessation in the postpartum period. The median time to breastfeeding cessation was 17 weeks shorter for women with HDP than those without HDP. Evidence of modification by race and ethnicity was identified for the association between HDP and breastfeeding cessation.

Despite a 2022 study of nearly 1418 women demonstrating no difference in prenatal breastfeeding intentions by HDP diagnosis,^55^ our study suggests a negative association between HDP and breastfeeding outcomes. Our findings are consistent with the few previously published observational studies that have shown shorter breastfeeding durations among women with HDP compared with those without.^29,54,55,56^ Most of studies were conducted outside the US,^29,54,55^ apart from 2 that used 2004-2015 data from CDC PRAMS. Burgess et al^56^ examined 5285 women in Illinois from 2012 to 2015 and found that individuals with HDP were less likely to ever breastfeed or to provide pumped breast milk to their infant (82% vs 87%; *P* < .001) or breastfeed for at least 8 weeks post partum (48% vs 55%; *P* = .002) compared with those without HDP. Those with HDP were more likely to stop breastfeeding for maternal illness or medical reasons (11.2% vs 6.3%; *P* = .002).^56^ Mulready-Ward and Sackoff^57^ examined 2004-2007 data from 7 PRAMS sites (raw number, 34 385) and showed that among preterm deliveries, HDP was associated with higher odds of breastfeeding for fewer than 8 weeks (OR, 1.34; 95% CI, 1.06-1.69).

Potential hypotheses for augmented or unique breastfeeding barriers experienced by dyads with HDP include hypertensive medications motivating appropriate or inappropriate recommendations to not breastfeed, as well as higher risks of dyadic separation for medical intervention and medical comorbidities associated with lower breastfeeding initiation,^45,46,47,50^ including cesarean delivery,^68^ obesity, and diabetes,^69^ and delayed secretory activation (ie, delayed lactogenesis II).^55,70,71,72^ Evidence of physiologic mechanisms to explain worse breastfeeding outcomes among women with HDP and other cardiometabolic disease is emerging.^72,73,74^ However, 2 observational studies did not observe a meaningful association between HDP and individuals’ attainment of a full milk supply after premature delivery.^75,76^ Altogether, these findings suggest the potential for breastfeeding interventions focused on achieving secretory activation in the immediate perinatal period to promote better breastfeeding outcomes for women with HDP. Such interventions could include antenatal milk expression^70^ or improved support with direct (if dyad united) and indirect (if dyad separated) breastfeeding in the first hours to days post partum.

### Public Health Implications

Women with HDP experience higher long-term risk of cardiovascular disease, including myocardial infarction, ischemic heart disease, and stroke, among others.^11,12,13,14,15^ Within our sample, HDP was associated with a higher hazard of breastfeeding cessation and shorter median duration. These findings cause concern, as breastfeeding duration directly relates to the degree of cardiometabolic benefit for the lactating woman.^21,22,23,24,25,26,27,28,29^ The duration of breastfeeding necessary to maximize maternal cardiovascular benefit is not clear. Observational studies have found cardiometabolic benefit after 4 months^28^ and 6 months^21,23,29^ of breastfeeding, although enhanced benefit has also been appreciated 12 months or later.^21,22,24,26^ These cardioprotective benefits have been observed as far as 7 to 18 years after breastfeeding exposure.^23,24,27,28,29^ Individuals with HDP may experience augmented cardiometabolic protection from breastfeeding. A 2023 study of 3598 women^29^ found a higher protective association between breastfeeding and cardiometabolic health among women with HDP compared with those without, with the strongest benefit seen among women who breastfed 6 to 9 months post partum.

We observed the highest prevalence of HDP among women younger than 18 and older than 34 years. The high prevalence of HDP among younger women could allude to a well-described rise in cardiometabolic disease prevalence among US youths^77,78^ and/or allude to possible confounding, as individuals who experience social or economic marginalization may be more likely to experience a teenage pregnancy^79,80^ and HDP, as observed in our descriptive analyses. A 2024 study of more than 14 000 children and young adults aged 10 to 24 years from New York City^51^ found that individuals with prediabetes in the preconception period had an 18% higher risk for pregnancy hypertension and preterm delivery. Suggestive of possible intergenerational sequalae of HDP, long-term associations between intrauterine HDP exposure and poorer offspring cardiovascular health in adolescence^16,17^ and adulthood^19^ have been observed. With 1 in 5 school-aged US children considered to have obesity,^81^ there is a growing urgency to develop, implement, and examine strategies to optimize cardiometabolic protection to infants and pregnant young women at high risk of future cardiometabolic disease. Such strategies could include breastfeeding promotion, as breastfeeding has been associated with a 33% lower risk of infant mortality^31^ and lower long-term risk of hypertension, obesity, and diabetes, to both women and their infants.^35^

### Promoting Health Equity

US populations with the highest HDP prevalence^1^ also have the lowest breastfeeding rates,^63^ including American Indian or Alaska Native and non-Hispanic Black women. These populations disproportionately miss the cardiometabolic and mortality protection associated with breastfeeding despite having higher risk. In our sample, HDP was most prevalent among women with Medicaid insurance and who were American Indian or Alaska Native (21.0%) or non-Hispanic Black (23.0%), aligned with findings from the 2022 CDC report on HDP.^1^ American Indian or Alaska Native and non-Hispanic Black dyads also have the highest risk of maternal mortality, with 12.8% and 8.2% of deaths of women in these populations, respectively, attributed to hypertension.^6,7^ Infants from these racial and ethnic populations have a 1.7 (American Indian or Alaska Native) and 2.4 (non-Hispanic Black) times higher mortality risk than non-Hispanic White infants.^9^ Several factors are likely to influence racial and ethnic disparities observed for HDP and breastfeeding outcomes, including differences in access to and quality of obstetric or lactation care,^82,83,84,85,86,87,88^ rates of postpartum depression,^89,90^ and discrimination and/or mistreatment during care, as 31% of American Indian or Alaska Native women and 40% of non-Hispanic Black women report discrimination during their maternity care.^1,91,92,93^ Our finding that race and ethnicity modified the association between HDP and breastfeeding cessation, with American Indian or Alaska Native and non-Hispanic Black women having some of the highest hazards of cessation among racial and ethnic groups, suggests that HDP may contribute to the observed disparities in breastfeeding rates between US racial and ethnic groups. To advance health equity, next studies should identify the biopsychosocial mechanisms that underlie the association between HDP and breastfeeding initiation and duration, then develop targeted strategies to support breastfeeding among dyads with HDP, perhaps tailored specifically to groups with the highest risk of HDP and lowest breastfeeding rates. Such strategies have the potential to more equitably provide cardiometabolic benefit to communities with the lowest breastfeeding rates^63,64^ and highest cardiometabolic risk.^1^

### Limitations

This study’s limitations include the potential for recall, response, and social desirability bias, all considered a risk when using retrospective, self-reported survey data. Although the cross-sectional design of our analysis limits our ability to assess causation or directionality in the association between HDP and breastfeeding outcomes, it provides important descriptive data on which to design future analyses aimed at assessing causality and directionality in this association. We were unable to assess gender differences due to the lack of questions assessing gender in PRAMS. Additionally, we were unable to examine specific populations of Asian or Pacific Islander women due to small sample sizes from individual racial and ethnic subgroups included in this variable (Chinese, Filipino, Hawaiian, Japanese, and Other Asian). Given the high hazard of breastfeeding cessation found for Asian or Pacific Islander women with HDP in our stratified analyses, future research is needed to examine if and to what extent the association between HDP and breastfeeding cessation differs between specific populations of Asian and Pacific Islander races and ethnicities.

Several well-documented barriers to breastfeeding not assessed in PRAMS could have influenced breastfeeding outcomes for our study’s participants, including maternal self-efficacy and prior breastfeeding experience, social support, workplace protections, and access to lactation support resources.^94,95,96,97^ Future studies should examine possible mechanisms to explain the association between HDP and breastfeeding identified in this study, how the severity of HDP (chronic or gestational hypertension vs preeclampsia vs eclampsia) influences this association, and how the implementation of evidence-based strategies known to improve breastfeeding outcomes, such as lactation support provided by peer counselors or community health workers, lactation consultants, and doulas, could be modified to address the unique barriers experienced by women with HDP.^98,99,100,101,102,103^

## Conclusions

Our cross-sectional study demonstrates that US women with HDP had higher odds of never breastfeeding, and for those who did initiate breastfeeding, a shorter median breastfeeding duration and higher hazard of breastfeeding cessation compared with women without HDP, with evidence of modification by race and ethnicity. Further analyses could inform tailored approaches to promote breastfeeding for dyads experiencing higher cardiometabolic risk and promote health equity for US women and infants.

## Notes

Supplement 1. **eTable.** Assessment of Potential Response Bias: A Comparison of Participants and Nonparticipants by Maternal-Infant Covariates

Supplement 2.
Data Sharing Statement
