# Supplementary material for: Hypertensive Disorders of Pregnancy and Breastfeeding Among US Women
Source: JAMA Netw Open. 2025 Jul 18;8(7):e2521902. doi: 10.1001/jamanetworkopen.2025.21902 (PMC12274979; doi:10.1001/jamanetworkopen.2025.21902)
Supplement: Supplement 1. — eTable. Assessment of Potential Response Bias: A Comparison of Participants and Nonparticipants by Maternal-Infant Covariates [file jamanetwopen-e2521902-s001.pdf]

Supplementary Online Content

Nardella D, Canavan ME, Taylor SN, Sharifi M. Hypertensive disorders of pregnancy and breastfeeding among US women. *JAMA Netw Open.* 2025;8(7):e2521902. doi:10.1001/jamanetworkopen.2025.21902

eTable. Assessment of Potential Response Bias: A Comparison of Participants and Nonparticipants by Maternal-Infant Covariates

This supplementary material has been provided by the authors to give readers additional information about their work.

© 2025 Nardella D et al. *JAMA Network Open*.

eTable. Assessment of Potential Response Bias: A Comparison of Participants and Nonparticipants by Maternal-Infant Covariates

	**Excluded Participants** Raw N (col %)[a] N=22,995 (10.2)	**Included Participants** Raw N (col %)[a] N= 205,247 (89.9)	**P value**
**Maternal Age (years)**	N=22,988		
<18	237 (1.0)	2,441 (1.2)	<0.001
18-24	4,295 (18.7)	43,936 (21.4)	
25-29	6,410 (27.9)	58,847 (28.7)	
30-34	7,046 (30.7)	61,332 (29.9)	
≥ 35	5,000 (21.8)	38,691 (18.9)	
**Insurance at Delivery**	N=21,487		
Medicaid	8,905 (41.4)	88,114 (42.9)	<0.001
Private	9,794 (45.6)	104,343 (50.8)	
Self-pay	1,816 (8.5)	4,808 (2.3)	
Other	972 (4.5)	7,982 (3.9)	
**Income, US $**	N=21,693		
Less than 20,000	5,442 (25.1)	52,571(25.6)	<0.001
20,001-40,000	4,346 (20.0)	39,999 (19.5)	
40,001-60,000	2,654 (12.2)	24,242 (11.8)	
60,001-85,000	2,311 (10.7)	20,756 (10.1)	
Over 85,000	4,935 (22.8)	51,019 (24.9)	
Not Reported	2,005 (9.2)	16,660 (8.1)	
**Race and Ethnicity**	N=17,344		
American Indian or Alaska Native	714 (4.1)	8,144 (4.0)	<0.001
Asian or Pacific Islander	1,519 (8.8)	14,232 (6.9)	
Black	3,054 (17.6)	36,143 (17.6)	
Hispanic	3,549 (20.5)	40,326 (19.7)	
White	7,182 (41.4)	95,328 (46.5)	
Multiracial	1,178 (6.8)	9,904 (4.8)	
Other	148 (0.9)	1,170 (0.6)	
**Women, Infants, and Children (WIC) Enrollment**	N=19,899		
Enrolled	12,984 (65.3)	129,162 (62.9)	<0.001
Not Enrolled	6,915 (34.8)	76,085 (37.1)	
**Marital Status**	N=22,833		
Married	13,907 (60.9)	122,869 (59.9)	0.002
Not married	8,926 (39.1)	82,378 (40.1)	
**Education Level**	N=21,177		
12 years or less	7,807 (36.9)	72,758 (35.5)	<0.001
More than 12 years	13,370 (63.1)	132,489 (64.6)	
**First Child**	N=17,982		

© 2025 Nardella D et al. *JAMA Network Open*.

Yes<br>No	17,651 (98.2)<br>331 (1.8)	201,729 (98.3)<br>3,518 (1.7)	0.21
**Singleton Pregnancy**	N=18,055		
Yes<br>No	17,337 (96.0)<br>718 (4.0)	198,374 (96.7)<br>6,873 (3.4)	<0.001
**Infant Birth Weight**	N=22,811		
Very Low (<1500 grams)<br>Low (<2500 grams)<br>Normal (2500-4000 grams)<br>Large (>4000 grams)	873 (3.8)<br>4,173 (18.3)<br>16,030 (70.3)<br>1,735 (7.6)	5,490 (2.7)<br>35,195 (17.2)<br>150,624 (73.4)<br>13,938 (6.8)	<0.001
**Infant Sex**	N=22,989		
Female<br>Male	11,583 (50.4)<br>11,406 (49.6)	103,086 (50.2)<br>102,161 (49.8)	0.65
**Premature Delivery**	N=22,793		
Yes (<37 weeks)<br>No (37+ weeks)	4,128 (18.1)<br>18,665 (81.9)	34,721 (16.9)<br>170,526 (83.1)	<0.001
**Infant Length of Hospital Stay**	N=19,179		
<3 days<br>3-5 days<br>>5 days	9,741 (50.8)<br>6,142 (32.0)<br>3,296 (17.2)	112,251 (54.7)<br>63,915 (31.1)<br>29,081 (14.2)	<0.001
**Maternal Body Mass Index (BMI)**	N=18,362		
BMI < 30<br>BMI ≥ 30	13,467 (73.3)<br>4,895 (26.6)	144,239 (70.3)<br>61,008 (29.7)	<0.001
**Smoking Status**	N=20,560		
Smoke<br>No Smoke	1,603 (7.8)<br>18,957 (92.2)	15,299 (7.5)<br>189,948 (92.6)	0.08
**Diabetes Before or During Pregnancy**	N=21,598		
Yes<br>No	2,688 (12.5)<br>18,910 (87.6)	27,048 (13.2)<br>178,199 (86.8)	0.002
**Depression Before or During Pregnancy**	N=22,880		
Yes<br>No	2,414 (10.6)<br>20,466 (89.5)	20,903 (10.2)<br>184,344 (89.8)	0.08
**Cesarean Section Delivery**	N= 22,835		
Yes<br>No	6,914 (30.3)<br>15,921 (69.7)	68,546 (33.4)<br>136,701 (66.6)	<0.001

[a] Columns may not add to 100 due to rounding.

© 2025 Nardella D et al. *JAMA Network Open*.
