# Supplementary material for: Hypertensive Disorders of Pregnancy and Breastfeeding Among US Women
Source: JAMA Netw Open. 2025 Jul 18;8(7):e2521902. doi: 10.1001/jamanetworkopen.2025.21902 (PMC12274979; doi:10.1001/jamanetworkopen.2025.21902)
Supplement: Supplement 2. — Data Sharing Statement [file jamanetwopen-e2521902-s002.pdf]

Data Sharing Statement

Nardella. Hypertensive Disorders of Pregnancy and Breastfeeding Among US Women. *JAMA Netw Open*. Published July 18, 2025. doi:10.1001/jamanetworkopen.2025.21902

Data
Data available: No

Additional Information
Explanation for why data not available: This data is publicly available through the CDC Pregnancy Risk Assessment Monitoring System.
